# DAMPs and radiation injury

**DOI:** 10.3389/fimmu.2024.1353990

**Published:** 2024-01-25

**Authors:** Satoshi Yamaga, Monowar Aziz, Atsushi Murao, Max Brenner, Ping Wang

**Affiliations:** ^1^ Center for Immunology and Inflammation, The Feinstein Institutes for Medical Research, Manhasset, NY, United States; ^2^ Departments of Surgery and Molecular Medicine, Zucker School of Medicine at Hofstra/Northwell, Manhasset, NY, United States

**Keywords:** DAMPs, radiation, necrosis, apoptosis, eCIRP, HMGB1

## Abstract

The heightened risk of ionizing radiation exposure, stemming from radiation accidents and potential acts of terrorism, has spurred growing interests in devising effective countermeasures against radiation injury. High-dose ionizing radiation exposure triggers acute radiation syndrome (ARS), manifesting as hematopoietic, gastrointestinal, and neurovascular ARS. Hematopoietic ARS typically presents with neutropenia and thrombocytopenia, while gastrointestinal ARS results in intestinal mucosal injury, often culminating in lethal sepsis and gastrointestinal bleeding. This deleterious impact can be attributed to radiation-induced DNA damage and oxidative stress, leading to various forms of cell death, such as apoptosis, necrosis and ferroptosis. Damage-associated molecular patterns (DAMPs) are intrinsic molecules released by cells undergoing injury or in the process of dying, either through passive or active pathways. These molecules then interact with pattern recognition receptors, triggering inflammatory responses. Such a cascade of events ultimately results in further tissue and organ damage, contributing to the elevated mortality rate. Notably, infection and sepsis often develop in ARS cases, further increasing the release of DAMPs. Given that lethal sepsis stands as a major contributor to the mortality in ARS, DAMPs hold the potential to function as mediators, exacerbating radiation-induced organ injury and consequently worsening overall survival. This review describes the intricate mechanisms underlying radiation-induced release of DAMPs. Furthermore, it discusses the detrimental effects of DAMPs on the immune system and explores potential DAMP-targeting therapeutic strategies to alleviate radiation-induced injury.

## Introduction

The increasing threat of unexpected exposure to ionizing radiation implicates the urgent unmet need for developing medical countermeasures. The global communities must be united to confront a broad range of nuclear threats. These encompass the detonation of sophisticated and improvised nuclear weapons, potential terrorist deployment of radiologic dispersal devices (i.e., dirty bombs), attacks on nuclear power plants, and the occurrence of accidents at these facilities caused by technical failure or natural disasters as happened in Chernobyl, Ukraine in 1986 and Fukushima, Japan in 2011 (1–4). Exposure to high doses of radiation can cause serious health problems known as acute radiation syndrome (ARS), consisting of three major sub-syndromes, hematopoietic (H), gastrointestinal (GI), and neurovascular (NV) ARS (5, 6). Medical countermeasures for ARS are classified into radioprotectors administered before radiation exposure, radiation mitigators administered shortly after radiation exposure and before symptoms appear, and radiation therapeutics administered after symptoms of radiation exposure appear (7). At the moment, the U.S. Food and Drug Administration (FDA) has approved two radioprotectors to be used in the context of radiotherapy: the free radical scavenger Amifostine and the recombinant human keratinocyte growth factor Palifermin (8). The FDA has also approved six radiomitigating medical countermeasures: the granulocyte colony-stimulating factors (G-CSF) Neupogen (filgrastim), Neulasta (pegfilgrastim), Udenyka (pegfilgrastim-cbqv), and Stimufend (pegfilgrastim-fpgk), the granulocyte-macrophage colony-stimulating factor (GM-CSF) Leukine (sargramostim), and thrombopoietin receptor agonist Nplate (romiplostim) (9). These radiomitigators are not approved as radioprotectors and only mitigate hematopoietic dysfunction to some extent. There are no FDA-approved medical countermeasures available for GI-ARS or NV-ARS. The major mechanisms of medical countermeasures currently under development include free radical scavenging, DNA damage reduction, DNA repair promotion, apoptosis inhibition, and GI recovery, in addition to the promotion of hematopoiesis (10, 11).

Exposure to ionizing radiation induces the release of damage-associated molecular patterns (DAMPs) by DNA damage and several types of cell death (12). A growing body of evidence suggests that DAMPs play pro-inflammatory roles and are associated with disease severity and organ damage in several diseases (13, 14). DAMPs hold the potential to function as mediators that exacerbate radiation-induced organ injuries, ultimately worsening survival outcomes. This review aims to comprehensively outline the mechanisms underlying radiation injury, with a particular focus on the involvement of DAMPs. Furthermore, we delve into potential therapeutic interventions aimed at mitigating radiation-induced DAMPs and their associated consequences.

## Acute radiation syndrome (ARS)

The hematopoietic system emerges as the most vulnerable organ to ARS, with discernible symptoms of H-ARS manifesting following exposure to total body irradiation (TBI) exceeding 2 gray (Gy) in humans (15). Depending on the dose of ionizing radiation, death of hematopoietic stem and progenitor cells and apoptosis of lymphocytes occur (16). Notably, lymphopenia becomes apparent within 6-24 hours after radiation exposure (17). Subsequent to this initial event of the loss of lymphocytes, neutropenia, thrombocytopenia, and anemia ensue, depending on the radiation dose, based on the half-life of these cells and cell fragments in the circulation (18). GI-ARS generally occurs in individuals following exposure to TBI exceeding 5 Gy (19). Radiation exposure damages progenitor cells in the crypts and differentiated cells in the villi cannot turn over due to the loss of the progenitor and stem cells (20, 21). Injury to the intestinal mucosal barrier results in the infiltration of antigens and bacteria into the intestinal wall from the lumen. This breach gives rise to various complications, including the onset of secretory diarrhea resulting in severe protein loss and electrolyte dysfunction, bacterial translocation from the intestinal lumen into the circulation, and intestinal hemorrhage (22). These complications collectively contribute to the lethal sepsis and bleeding along with neutropenia and thrombocytopenia due to H-ARS (23). NV-ARS occurs after TBI with over 10 Gy in humans (19). Damage to the central nervous system involves disruptions in capillary circulation, blood-brain barrier integrity, interstitial edema, and the onset of acute inflammation. NV-ARS results in death within a few days of radiation exposure (19). Human data following radiation exposure indicates that the LD_50/60_, estimating the radiation dose causing lethality in 50% of a population within 60 days, ranges from 3.3-4.5 Gy without medical management to 6-7 Gy with medical intervention, including antimicrobial agents and blood transfusions (17, 23). In addition to ionizing radiation, nuclear explosions also release energy in the form of heat and shock waves (3). Thus, in situations involving nuclear explosions, 60-70% of victims may suffer from a radiation-combined injury (RCI) that includes burns and trauma (24). As a result, RCI can be lethal at low radiation doses due to infections (and possibly increased release of DAMPs, as described later in this review) in patients with burns and trauma (22, 25).

Oxidative stress causes DNA damage by reactive oxygen species (ROS) generated after the ionization of water in the cells. Ionizing radiation can also induce direct DNA damage including single- and double-strand breaks (26). The DNA damage response induces a reversible cell cycle arrest to give cells time to repair damage (12). When DNA damage cannot be resolved by the repair process, the DNA damage response converts into a signal pathway of apoptotic cell death or senescence, most often through p53-dependent mechanisms (27). Mitotic catastrophe can occur when cells undergo DNA damage during mitosis or enter mitosis with DNA damage. Mitotic catastrophe causes the formation of cells with multi-micronuclei or two nuclei (28). These cells are unable to complete the cell cycle, ultimately leading to their entry into senescence or their demise through apoptosis or necrosis (27). Exposure to ionizing radiation can affect not only nuclear DNA, but also other cellular organelles including mitochondria, Golgi apparatus, and lysosomes (29). Mitochondria serve as the primary generators of ROS within the cells. Radiation-induced mitochondrial DNA damage induces ROS generation, leading to a decrease in mitochondrial membrane potential and inducing mitochondria outer membrane permeabilization (MOMP) (29). The subsequent release of cytochrome c and activation of caspase-9 lead to apoptosis (30).

Recent studies have unveiled non-apoptotic signaling pathways of programmed cell death such as necroptosis, pyroptosis, and ferroptosis as additional forms of ionizing radiation-induced cell death. For instance, oxidative stress induced by ionizing radiation activates the inflammasome pathway, causing pyroptosis and contributing to organ damage (31, 32). Necroptosis in the intestine has been shown to contribute to mortality after lethal radiation exposure (33). Furthermore, the accumulation of ROS resulting from radiation exposure has been linked to the induction of ferroptosis, correlating with H-ARS, GI-ARS, and radiation-induced lung injury (34–36).

## Damage-associated molecular patterns (DAMPs)

The immune system initiates an immune response against invasive pathogens based on the distinction of ‘self’ from ‘non-self’ (37). Innate immune cells express pattern recognition receptors, such as Toll-like receptors (TLRs), recognizing pathogen-associated molecular patterns (PAMPs) (37). Pathogen-independent immune responses are also initiated by recognition of DAMPs released from stressed or damaged cells (38). DAMPs activate innate immune cells such as neutrophils, macrophages, and dendritic cells (39). Consequently, various cytokines and chemokines are released, activating adaptive immune responses (40). DAMPs also activate non-immune cells such as epithelial cells, endothelial cells, and fibroblasts, leading to the release of inflammatory mediators (37). Typical endogenous DAMPs include extracellular cold-inducible RNA-binding protein (eCIRP), high mobility group box 1 (HMGB1), histones, heat shock proteins (HSPs), ATP, and nuclear and mitochondrial DNA (mtDNA), extracellular RNA (exRNA), and uric acid (38). The major DAMP-recognizing pattern recognition receptors include TLRs, NOD-like receptors (NLRs), RIG-I-like receptors, and C-type lectin receptors (41). Following the recognition of DAMPs, TLRs interact with myeloid differentiation factor 88 and TIR-domain-containing adaptor-inducing beta interferon to phosphorylate downstream mitogen-activated protein kinases, leading to activation of the transcription factors, activator protein-1 and nuclear factor kappa B (NF-κB) (37). DAMPs also transduce signals through the receptor for advanced glycation end products (RAGE), triggering receptor expressed on myeloid cells-1 (TREM-1), G-protein-coupled receptors, transient receptor potential, and P2X7 receptor channels (37).

The release of DAMPs has been observed in radiation injury and a number of other diseases including sepsis, trauma, ischemia-reperfusion injury, brain injuries, and radiation exposure (39, 42–45). In sepsis, host recognition of DAMPs is a critical contributor to an excessive immune response. Circulating DAMPs correlate with disease severity, and their inhibition has been shown to improve outcomes in experimental models of sepsis (38, 39). In severe trauma, the escalation of the innate immune response due to exposure to DAMPs leads to coagulopathy and excessive inflammation, resulting in multiple organ dysfunction, such as intestinal barrier dysfunction and acute lung injury (46). In intestinal ischemia-reperfusion injury, circulating mtDNA correlates with inflammatory responses and contributes to intestinal injury (43). Excessive inflammatory signaling induced by DAMPs can exhaust hematopoiesis and induce dysfunction of hematopoietic stem cells (47). Furthermore, the DAMPs-mediated neuroinflammation after traumatic brain injury, subarachnoid hemorrhage, and cerebral ischemia exacerbates secondary damage of the central nerve system by promoting cerebral edema or triggering vascular spasm and microthrombi (48). Given that these clinical complications are frequently observed in ARS, it is evident that the release of DAMPs can exacerbate the outcomes in ARS.

## DAMP release after radiation injury

Radiation-induced cell damage can signal the immune system to potential danger through the release of DAMPs, akin to the molecular pattern recognition seen in microorganisms. Inflammation resulting from an excess of DAMP release has the potential to worsen radiation-induced tissue and organ damage, emphasizing the significance of DAMP modulation as potential mitigation for radiation injury (49). Here, we provide a summary of the major DAMPs released during radiation injury, and explore interventions aimed at enhancing outcomes (**Table 1**).

**Table 1:** Targeting DAMPs in radiation injury.

DAMPs	Radiation model	Interventions	Outcomes	References
eCIRP	6.5 Gy TBI, X-ray, C57BL/6 mice	CIRP knockout/TREM-1 knockout	Improves overall survival rate	(49)
HMGB1	6.5 Gy PBI, X-ray, C57BL/6 mice	Glycyrrhizin	Decreases pro-inflammatory cytokine production and mitigates intestinal injury	(50)
	20 Gy PBI, X-ray, C57BL/6 mice	Glycyrrhizin	Decreases pro-inflammatory cytokine production and mitigates lung injury	(51)
mtDNA	6 Gy TBI, γ-ray, C57BL/6 mice	VDAC1 inhibitor (mtDNA release inhibition)	Decreases type I-IFN mRNA expression and mitigates hematopoietic injury	(52)
exRNA	10 Gy TBI, γ-ray, BALB/c mice	TLR3/double-stranded RNA complex inhibitor	Mitigates intestinal injury and improves overall survival rate	(53)
	9 Gy TBI, γ-ray, BALB/c mice	Exosome inhibitor	Improves overall survival rate	(54)
SAA	2-8 Gy TBI/PBI, X-ray, C57BL/6 mice	N/A	Useful for radiation biodosimetry at 24 hours after irradiation	(55, 56)
	12, 14 Gy PBI, X-ray, C57BL/6 mice	N/A	Useful for radiation biodosimetry at 3 and 4 days after irradiation	(57)

CIRP, cold-inducible RNA-binding protein; DAMPs, damage-associated molecular patterns; exRNA, extracellular RNA; HMGB1, high mobility group box 1; IFN, interferon; mtDNA, mitochondrial DNA; SAA, serum amyloid A; PBI, partial body irradiation; TBI, total body irradiation; TLR, Toll-like receptor; TREM-1, triggering receptor expressed on myeloid cells-1; VDAC, voltage-dependent anion channel; N/A, not available.

### Extracellular cold-inducible RNA-binding protein (eCIRP)

Intracellular CIRP is an RNA chaperone that functions as a cellular stress response protein (58). Once outside of the cell, eCIRP plays an important role as an endogenous inflammatory mediator acting as a new DAMP in radiation injury, sepsis, hemorrhagic shock, and ischemia-reperfusion injury (58, 59). eCIRP induces the production of proinflammatory cytokines and chemokines in macrophages via the TLR4/MD2 complex and NF-κB signaling axis. Accordingly, it contributes to tissue damage by upregulating ICAM-1 expression and promoting excessive NET formation in neutrophils (58, 60). eCIRP can also bind to TREM-1, worsening organ damage and prognosis by promoting inflammation (61). eCIRP has been shown to suppress the bacterial phagocytosis of macrophages by promoting the formation of STAT3-βPIX complex and suppressing the activation of Rac1 (62). We have recently demonstrated that high-dose ionizing radiation induces eCIRP release in the peritoneal cavity of irradiated mice and in the cell culture supernatant of irradiated mouse peritoneal cells (49). Moreover, the radiation-induced eCIRP release upregulated the expression of TREM-1 on macrophages, and mice knocked out for CIRP or TREM-1 had improved 30-day survival rates after 6.5 Gy TBI (49). Therefore, targeting the eCIRP-TREM-1 axis holds promise as a therapeutic strategy for post-radiation injury, potentially attenuating lethal infections and sepsis (49).

### High mobility group box 1 (HMGB1)

HMGB1 is a nuclear protein that acts as a DNA chaperone (63). As a DAMP, HMGB1 binds to pattern recognition receptors, including RAGE, TLR2, TLR4, TLR9, and TREM-1, to induce inflammatory responses (64, 65). In a murine model of sepsis, HMGB1, released from macrophages, has been shown to function as a late lethal mediator (66). In acute lung injury and hepatic ischemia-reperfusion injury models, administration of anti-HMGB1 antibody reduces lung neutrophil influx and pulmonary edema and mitigates liver damage (67, 68). Exposure to ionizing radiation increases in both cellular mRNA expression and serum protein levels of HMGB1 in a dose-dependent manner in experimental mice model (69). Glycyrrhizin, one of the inhibitors of HMGB1, reduced the release of serum inflammatory cytokines in abdominal irradiated mice (6.5 Gy) through the downregulation of the HMGB1/TLR4 signaling pathway and significantly attenuated intestinal injury (50). In thoracic irradiated mice, glycyrrhizin also reduced inflammatory cytokines in the bronchoalveolar lavage fluid and mitigated radiation-induced lung injury by inhibiting downstream transcription factors related to the HMGB1/TLR4 axis (51).

### Mitochondrial DNA (mtDNA)

mtDNA is susceptible to ionizing radiation compared to nuclear DNA due to the lack of protective histones and limited repair mechanism (29). Mitochondrial dysfunction resulting from failure of DNA repair leads to the release of mitochondrial components including cytochrome c, ATP, mtDNA, and mtRNA (29). The mtDNA in the cytosol is recognized by the cyclic GMP-AMP synthase (cGAS) to activate the stimulator of interferon genes (STING) signaling pathway, resulting in the production of type I interferons (IFNs) (65, 70). Excessive production of type I IFNs can cause exhaustion in hematopoietic stem cells (71, 72). In the 6 Gy TBI model, mtDNA released into the cytosol has been reported to activate the cGAS-STING pathway and induced type I IFN production, and inhibition of the mtDNA release ameliorated hematopoietic tissue injury (52). The mtDNA released into the cytoplasm can also bind to and activate the NLRP3 inflammasome, which leads to the release of inflammatory cytokines such as IL-1β and IL-18 followed by pyroptosis (31). Previous studies have shown that pyroptosis in intestinal cells occurred through NLRP3 activation, inducing intestinal injury in the abdominal irradiation model (73, 74). These studies suggest that mtDNA exacerbates ARS pathophysiology and could be a potential therapeutic target for GI-ARS by mitigating pyroptosis and inflammation.

### Extracellular RNA (exRNA)

exRNA from host cells is carried along microvesicles and exosomes which protect exRNA from enzyme degradation (75). exRNA acts as a pivotal signaling molecule to mediate communication between cells, but can also serve as DAMPs (75, 76). TLR3 has been known to recognize not only double strand RNA released from virus-infected cells but also endogenous exRNA released from necrotic cells, promoting inflammation and organ dysfunction in autoimmune diseases and sepsis (77–79). It has been shown that large exRNA released from the host small intestine after ionizing radiation exposure induced extensive crypt cell death via TLR3 and inhibition of exRNA binding to TLR3 improved survival in 10 Gy TBI by preventing GI-ARS (53). In the mouse model after 9 Gy TBI, the release of microRNAs (miRNAs) via exosomes worsened intestinal injury by promoting apoptosis and DNA damage of intestinal cells (54). These findings implicate a potential detrimental role for exRNA in ARS.

### Serum amyloid A (SAA)

In a radiological event, mass screenings are necessary to triage exposed and non-exposed individuals and to determine the severity of the radiation dose to the exposed population (55). Established biomarkers for estimating radiation dose in exposed individuals are quantification of chromosomal aberrations and γ-H2AX, a marker of DNA double-strand breaks, but these tests are only available in laboratories with trained personnel. Serum amyloid A (SAA) is one of the proteins associated with acute phase reactions resulting from inflammation, infection, trauma, or other events (80). SAA has also been reported as a radiation-responsive biomarker in TBI and partial-body irradiation (PBI) models (55–57, 81). In the mice, after 2-8 Gy TBI, SAA is significantly increased at 24 hours after irradiation compared to controls (55, 56). In the mouse PBI model of GI-ARS, SAA levels significantly increase 3 and 4 days after irradiation with 12 Gy and 14 Gy, respectively (57). Although the serum concentration of SAA increases within a few hours after irradiation and returns to the baseline within 7 days after irradiation, combinations of SAA with other biomarkers provide greater accuracy for the assessment of radiation injury especially, at the acute phase (56, 81).

## Mechanisms of DAMP release – passive pathways

**Figure 1:**
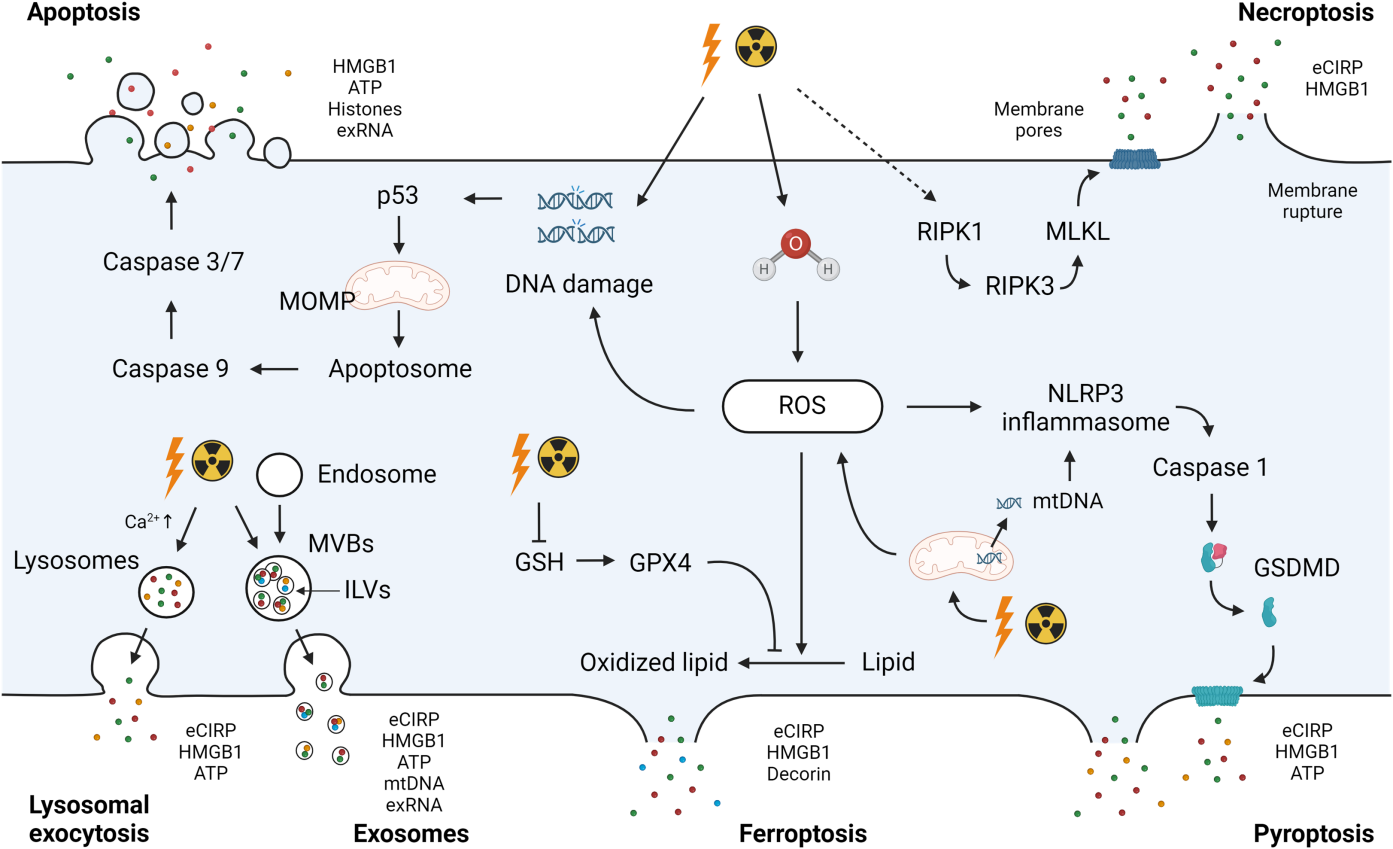
Radiation-induced DAMPs release. Exposure to ionizing radiation causes direct DNA damage or ROS-induced DNA damage to activate the intrinsic pathway of apoptosis. Mitochondrial damage by ionizing radiation amplifies ROS production and contributes to DNA damage. ROS accumulation after radiation exposure due to GPX4 impairment and GSH depletion induces the oxidation of polyunsaturated fatty acids in membrane phospholipids, leading to ferroptosis. Radiation-induced ROS leads to activation of the NLRP3 inflammasome. The NLRP3 inflammasome complex activates caspase-1 to cleave GSDMD and form a membrane pore with the GSDMD-N domain, inducing pyroptosis. Ionizing radiation causes the activation of RIPK1, which recruits RIPK3 to phosphorylate MLKL. Phosphorylated MLKL forms MLKL oligomers, leading to necroptosis. DAMPs including eCIRP, HMGB1, ATP, histones, exRNA, and mtDNA can be released as a passive release mechanism after these cell deaths. Mitochondrial dysfunction results in the release of mitochondrial components including mtDNA into the cytosol. Secretion mechanisms such as exocytosis in the forms of lysosomes and exosomes cause active release of DAMPs. DAMPs, damage-associated molecular patterns; ROS, reactive oxygen species; GPX4, glutathione peroxidase 4; GSH, glutathione; NLRP3, NLR family pyrin domain containing 3; GSDMD, Gasdermin D; RIPK, receptor-interacting serine/threonine-protein kinase; MLKL, mixed lineage kinase domain like pseudokinase; eCIRP, extracellular cold-inducible RNA-binding protein; HMGB1, high mobility group box 1; exRNA, extracellular RNA; mtDNA, mitochondrial DNA; MOMP, mitochondria outer membrane permeabilization; MVBs, multivesicular bodies; ILVs, intraluminal vesicles.

The pathways of DAMP release can be categorized into two primary pathways: i) passive release and ii) active release (38). Passive release is primarily linked to cell death processes, including apoptosis, necrosis/necroptosis, pyroptosis, and ferroptosis. Concurrently, DAMPs are actively released through secretion mechanisms such as exocytosis in the forms of lysosomes and exosomes (38). Below, we have outlined the universal approaches to DAMP release. Given that radiation injury is directly associated with these cell death events or initiates pathways relevant to the active release of DAMPs, it is reasonable to infer that these processes are linked to the mechanisms governing DAMPs release in ARS (**Figure 1**).

### Apoptosis

Apoptosis is a form of programmed cell death, initiated by two distinct p53-dependent signaling pathways: the extrinsic and intrinsic apoptosis (82). In the extrinsic receptor-mediated pathway, extracellular death receptors are activated by ligand binding and death-inducible signaling complex (DISC) is formed at the intracellular tail of the receptor to activate pro-caspase-8, or pro-caspase-10, with adapter proteins such as Fas-associated via death domain. The extrinsic apoptosis is also induced by dependence receptors, which mediate lethal signals in the absence of their ligand, and pro-caspase-9 is activated (83, 84). Activated caspase-8 (or caspase-10) and caspase-9 eventually activate executioner caspase-3 and caspase-7 to induce apoptosis (83). On the other hand, severe DNA damage induced by radiation mainly activates the intrinsic pathway (82). A transcription factor p53 induces pro-apoptosis Bcl family proteins including BAX, NOXA and PUMA (85, 86). These proteins mediate MOMP, resulting in the release of mitochondrial proteins into the cytosol (82). Cytochrome c binds to apoptotic peptidase-activating factor 1 and pro-caspase-9 to form apoptosomes that ultimately activate caspase-3 and caspase-7 via the activation of caspase-9 (12, 82). During apoptosis, DAMPs such as HMGB1, ATP, histones, and exRNA are released into the extracellular space (87–90).

### Necrosis/necroptosis

Necrosis is an accidental cell death caused by external stimuli such as physical and chemical stress and heat (91). In necrotic cell death, the increased cell expansion disrupts the plasma membrane. As a result, cytoplasmic contents and pro-inflammatory molecules are released into the extracellular space (92). DAMPs, including HMGB1, HSP, and ATP, are shown to be released from necrotic cells (91, 93, 94). Necroptosis is a form of programmed cell death dependent on the activation of receptor-interacting serine/threonine-protein kinase 1 (RIPK1). RIPK1 activation can be caused by the stimulation of tumor necrosis factor (TNF) receptor and other death receptors such as Fas. Activated RIPK1 recruits RIPK3 and this complex phosphorylates mixed lineage kinase domain-like pseudokinase (MLKL) (95). Phosphorylated MLKL forms MLKL oligomers and translocates to the plasma membrane, resulting in inducing membrane permeabilization and cell disruption (95). The inhibition of necroptosis has been shown to decrease HMGB1 release in an LPS-induced intestinal injury model, demonstrating the contribution of necroptosis to the release of HMGB1 (96). eCIRP has also been demonstrated to be released by necroptosis in a sepsis model (97).

### Pyroptosis

Pyroptosis is a lytic cell death process that allows the release of potential immunostimulatory molecules. This programmed cell death can be induced by the canonical and non-canonical pathways (98). In the canonical pathway, pathogen-derived molecules are recognized by inflammasomes. For example, cell stressors including bacteria, viruses, and DAMPs induce potassium efflux, leading to NIMA-related kinase 7 binding to NLR family pyrin domain containing 3 (NLRP3). Subsequently, NLRP3 oligomerization is induced to activate pro-caspase 1 through the adaptor protein ASC (apoptosis-associated speck-like protein containing a CARD) (99). Caspase-1 processes and activates pro-IL-1β and pro-IL-18 and cleaves Gasdermin D (GSDMD) to form a membrane pore with the GSDMD-N domain. In the non-canonical pathway, endotoxin from Gram-negative bacteria directly activates pro-caspase-4 or pro-caspase-5 in humans (or pro-caspase-11 in mice), which also cleave GSDMD. Small DAMPs such as eCIRP (17-19 kDa) are then released through GSDMD’s pores along with IL-1β and IL-18 (100, 101). The release of larger DAMPs, such as HMGB1, can ensue when irreversible GSDMD pores cause terminal cell rupture (102, 103).

### Ferroptosis

Ferroptosis is a form of programmed cell death characterized by the iron-dependent accumulation of lipid peroxidation (104). At the steady state, intracellular glutathione peroxidase 4 (GPX4) utilizes glutathione (GSH) to reduce lipid peroxidation generated by ROS. However, ROS accumulation due to the impairment of GPX4 and depletion of GSH causes the oxidation of polyunsaturated fatty acids in membrane phospholipids through an intracellular iron-dependent mechanism, leading to ferroptosis (83, 104). DAMPs released by ferroptosis include eCIRP, HMGB1, and decorin, a small leucine-rich proteoglycan of the extracellular matrix (105–107). Decorin, similar to HMGB1, binds to TLR4 and RAGE to induce pro-inflammatory signaling (107, 108).

## Mechanisms of DAMP release – active pathways

### Exosomes

Exosomes are nano-sized membrane vesicles released upon fusion of multivesicular bodies (MVBs) with the plasma membrane and contain a variety of proteins, lipids, and nucleic acids (109). The endosomal membranes in early endosomes invaginate to form intraluminal vesicles (ILVs) in the lumen of the vesicular organelles (110). ILVs mature into MVBs by the endosome sorting complex required for transporting (ESCRT)-dependent or ESCRT-independent mechanism. MVBs are then transported to the plasma membrane dependent on their interaction with actin and the microtubule cytoskeleton to be released into the extracellular environment as exosomes (110). Exosomal DAMPs include HMGB1, HSPs, ATP, histones, mtDNA, and eCIRP (111–116). Increased DAMPs in exosomes induce inflammation and exacerbate organ dysfunction in a plethora of disorders including sepsis (117).

### Lysosomal exocytosis

Lysosomal exocytosis is another mechanism of active release of DAMPs. After stimulation of a cell receptor, an increase in intracellular Ca^2+^ is detected by synaptotagmin, which is an intracellular calcium sensor on lysosomes. The lysosomes are mobilized to the microtubule organizing center to be associated with the kinesin and transported to the site of secretion (118). The lysosomes dock and fuse with the plasma membrane through soluble *N*-ethylmaleimide-sensitive factor attachment proteins receptor (SNARE) complex Rab proteins, leading to secretion of the soluble components (118, 119). HMGB1, ATP, and eCIRP have been shown to be released by lysosomal exocytosis (58, 120, 121).

## DAMPs in sepsis and multiple organ failure after radiation injury

Sepsis from enteric microorganisms in GI-ARS is the major cause of morbidity and mortality in radiation injury (19). The breakdown of the mucosal barrier due to disruption of the intestinal epithelium leads to severe secretory diarrhea and translocation of bacteria into the systemic circulation, resulting in severe sepsis (20). Furthermore, damage to the intestinal mucosa and sepsis of enteric origin occur even after TBI in the H-ARS dose range (122). DAMPs generated by the disruption of the intestinal mucosal barrier in sepsis are transported through the mesenteric lymphatics into the systemic circulation, developing multiple organ failure such as acute lung injury and further deteriorating intestinal barrier injury (123). In RCI with burn and trauma, the pathophysiological role of DAMPs remains unexplored. In severe burns, DAMPs are released from the injured tissue, inducing systemic inflammatory response (124). Immunological exhaustion due to the excessive inflammatory response leads to inhibition of the innate and acquired immune systems, resulting in increased susceptibility to bacterial infections not only from the skin but also from the lung or gut microbiota to cause severe sepsis (124, 125). Furthermore, DAMPs release in trauma is associated with the development of organ injuries such as acute lung injury and acute renal failure (126, 127). DAMPs are also involved in the activation of coagulation pathways, leading to coagulopathy during trauma (124). Additionally, DAMPs have been suggested to induce post-traumatic immunosuppression (42). In the context of RCI, DAMPs may play a critically detrimental role in multiple organ failure and compromised immune systems, thereby exacerbating the prognosis of the patients.

## Future perspectives

Medical countermeasures for ARS are currently limited and only available for H-ARS (128). We described DAMPs as exacerbating factors and potential therapeutic targets for ARS. Pharmacological strategies targeting DAMPs to inhibit immune responses include the uses of monoclonal antibodies, peptides, decoy receptors, and small molecules (129). CIRP-derived small peptides that inhibit eCIRP binding to the TLR4-MD2 complex and binding to TREM-1 have been developed and shown to attenuate sepsis, acute kidney injury, and hepatic ischemia-reperfusion injury (61, 130–132). A molecule that directly neutralizes eCIRP has also been identified to improve acute lung injury and survival in sepsis (133). Additionally, we have recently described an engineered oligopeptide that effectively promotes eCIRP clearance from the circulation leading to markedly improved outcomes in sepsis (134). Anti-HMGB1 antibodies have been to be effective in various experimental models such as abdominal sepsis and hemorrhagic shock (63, 135, 136). Small molecules that bind to and block HMGB1, such as glycyrrhizin, and decoy receptors that block HMGB1-RAGE signaling, such as soluble RAGE, also improved several inflammatory disease models (137, 138). exRNAs include non-coding RNA (ncRNA) such as miRNAs, long non-coding RNAs, and circular RNAs as well as messenger RNAs. Recently, miRNAs have been identified as potential biomarkers for various diseases, including radiation injury (139). In experimental radiation models, serum miRNA profiling has been shown to be able to identify exposure to radiation and predict the extent of ARS and the probability of survival, potentially facilitating timely intervention after radiation exposure (140, 141). The high stability and reproducibility of serum miRNAs make them attractive candidates as biomarkers of radiation injury (141). The function of specific ncRNAs as DAMPs in radiation injury remains unclear and may be the subject for future research. In the context of RCI resulting from nuclear explosions, more than half of the patients are accompanied by burns and trauma, resulting in more complicated and severe radiation injury (24). FDA-approved countermeasures for H-ARS alone have failed to improve survival in RCI (24). DAMPs released in burn and trauma injury have potential links with the mechanical injury, ischemia/reperfusion injury, metabolic acidosis, and hypoxia, culminating in multiple organ failure (124). These findings underscore the potential of DAMPs-targeting strategies in addressing the challenges posed by RCI.

## Conclusions

In conclusion, exposure to ionizing radiation causes direct DNA damage and oxidative stress, leading to cell death. The subsequent release of DAMPs has the potential to worsen ARS across various body compartments. However, there is a scarcity of studies specifically addressing DAMPs in the context of radiation injury. A more profound comprehension of the role of DAMPs in radiation injury could pave the way for the development of innovative therapeutic strategies to mitigate the impact of nuclear and radiological threats and accidents.

## Author contributions

SY: Data curation, Formal Analysis, Investigation, Methodology, Writing – original draft, Writing – review & editing. MA: Conceptualization, Funding acquisition, Methodology, Resources, Supervision, Validation, Writing – original draft, Writing – review & editing. AM: Investigation, Methodology, Writing – original draft, Writing – review & editing. MB: Formal Analysis, Investigation, Supervision, Visualization, Writing – review & editing, Funding acquisition. PW: Conceptualization, Funding acquisition, Project administration, Resources, Supervision, Writing – review & editing.
